# The language of crisis: spatiotemporal effects of COVID-19 pandemic dynamics on health crisis communications by political leaders

**DOI:** 10.1038/s41746-021-00554-w

**Published:** 2022-01-10

**Authors:** Benjamin J. Mandl, Ben Y. Reis

**Affiliations:** 1 Predictive Medicine Group, Computational Health Informatics Program, Boston Children’s Hospital, Boston, MA USA; 2 Washington University, St. Louis, MO USA; 3 Harvard Medical School, Boston, MA USA

**Keywords:** Epidemiology, Epidemiology, Health policy

## Abstract

In times of crisis, communication by leaders is essential for mobilizing an effective public response. During the COVID-19 pandemic, compliance with public health guidelines has been critical for the prevention of infections and deaths. We assembled a corpus of over 1500 pandemic-related speeches, containing over 4 million words, delivered by all 50 US state governors during the initial months of the COVID-19 pandemic. We analyzed the semantic, grammatical and linguistic-complexity properties of these speeches, and examined their relationships to COVID-19 case rates over space and time. We found that as COVID-19 cases rose, governors used stricter language to issue guidance, employed greater negation to defend their actions and highlight prevailing uncertainty, and used more extreme descriptive adjectives. As cases surged to their highest levels, governors used shorter words with fewer syllables. Investigating and understanding such characteristic responses to stress is important for improving effective public communication during major health crises.

## Introduction

During times of crisis, communication by leaders is essential for mobilizing an effective and coordinated public response. The COVID-19 pandemic presented leaders, and for the purposes of this study, specifically governors, with one of the greatest public health challenges in modern times. Communicating specific public health guidelines to the public and promoting their compliance has been central to meeting this challenge, as the actions and behaviors of individuals directly impacted pandemic spread and individual outcomes, including illness and death.

The specific words through which leaders choose to communicate can have a direct effect on public compliance with public health guidelines such as social distancing and mask-wearing. Prior studies have shown that people’s behavior can be significantly affected by the manner in which instructions are presented^1^, and that specific word choice is often designed to shape public opinion^2^. A recent study found that after Brazil’s president intentionally and publicly minimized the importance of social distancing and downplayed the risks of the COVID-19 pandemic, social distancing behaviors in areas with strong political support for the president decreased relative to areas with weaker political support for the president^3^.

Leaders employ diverse approaches to public communication. The words chosen by leaders have been found to be associated with a wide range of factors, including the leader’s political affiliations^4,5^, demographic characteristics^6^, personal linguistic style^7,8^ and emotional state^9^. Speeches can also be influenced by the dynamics of the unfolding crisis^9^ and the underlying characteristics of the local population.

Computer-based approaches to analyzing large corpora of texts provide a systematic quantitative approach to identifying salient patterns in the texts, which can in turn reveal deeper underlying phenomena. With the rise of computer technology and increased digitization of texts, corpus linguistic methods have been used to study a wide range of phenomena, including the evolution of language^10^, historical epidemiology^11^, historical positivity bias^12^, cultural shifts^13^, and political trends^14^. Applying these approaches to modern political speech, studies have variously examined the semantic content, grammatical properties^4,8,15^, and linguistic complexity^4,5,16^ of speeches by public leaders. Studies have found that the complexity of speeches declined during times of crisis^17–19^.

In this study, we assembled a corpus of over 1500 pandemic-related speeches delivered by governors of all 50 US states during the initial months of the COVID-19 pandemic. We set out to study the associations, across both space and time, between COVID-19 case rates and the semantic, grammatical and linguistic complexity properties of these political speeches. We sought to determine whether the spatial and temporal dynamics of the pandemic affected the topics that governors chose to speak about, as well as the manner in which they did so. We also investigated the relationships between pandemic intensity and characteristic linguistic markers of stress, which can undermine effective public communication strategies at the time they are needed most.

## Results

### Data Corpus

We assembled a corpus of 1515 speeches, containing 4,049,146 words, delivered by all 50 US state governors. Median speech length was 1984 words, with speech lengths ranging from 100 words to 12,557 words. The number of available speeches varied by state, and by month within each state (Table 1). Overall, the speeches were a mix of some pre-written statements along with spontaneous speech.

**Table 1:** Governor speeches available per US state.

State	Total Speeches	Total Words	Shortest Speech (Words)	Longest Speech (Words)	March	April	May	June	July
AK	51	52,166	162	2546	15	19	12	3	2
AL	9	13,494	419	2529	2	4	2	1	0
AR	32	47,530	341	3274	2	6	1	13	10
AZ	23	27,523	316	4046	9	6	2	5	1
CA	55	328,698	2749	10,825	6	24	11	8	5
CO	22	97,399	1512	8914	5	7	3	4	3
CT	13	22,530	663	3692	1	3	4	2	3
DE	22	53,700	459	4771	1	8	8	4	1
FL	46	214,454	244	9930	8	13	14	4	7
GA	12	41,662	490	7330	3	4	4	1	0
HI	29	24,463	455	1624	2	10	9	5	3
IA	30	41,422	395	3471	6	8	11	5	0
ID	20	20,614	267	3743	7	3	4	5	1
IL	33	127,521	1036	5734	8	14	10	1	0
IN	18	22,641	114	2994	3	2	7	5	1
KS	41	40,493	322	2279	8	18	7	7	1
KY	20	93,079	292	9037	2	6	2	5	5
LA	17	58,283	688	6723	3	7	1	4	2
MA	34	95,122	774	5481	5	11	4	11	3
MD	12	34,536	475	4209	2	7	1	1	1
ME	22	22,542	586	2273	4	5	7	4	2
MI	27	105,623	1002	5379	4	10	9	2	2
MN	28	80,813	156	7663	2	8	9	8	1
MO	52	45,099	219	2258	10	14	16	9	3
MS	53	57,970	334	3339	4	21	17	8	3
MT	25	36,206	511	3368	8	7	2	6	2
NC	15	36,373	1400	3432	5	4	4	1	1
ND	32	130,858	255	8994	10	9	6	5	2
NE	52	82,059	671	4653	8	19	19	4	2
NH	25	29,904	467	3289	0	6	7	10	2
NJ	38	252,005	675	9022	4	19	10	3	2
NM	21	55,552	366	9862	6	6	5	2	2
NV	14	21,094	100	3669	6	3	1	2	2
NY	91	486,000	1735	9313	15	29	26	17	4
OH	33	163,806	1200	12,507	8	13	6	3	3
OK	22	21,619	373	2015	7	9	4	1	1
OR	20	20,780	418	3147	8	3	3	4	2
PA	20	29,802	538	7735	6	2	5	6	1
RI	77	271,951	1713	6194	11	27	22	14	3
SC	21	30,453	327	3268	8	6	2	2	3
SD	19	21,423	370	4596	0	6	12	1	0
TN	12	20,604	335	3780	1	6	2	1	2
TX	27	87,555	499	6927	5	11	6	3	2
UT	26	72,548	815	6774	5	13	4	3	1
VA	30	91,391	567	4888	6	10	10	3	1
VT	34	29,231	442	2342	1	9	12	11	1
WA	24	68,510	840	6391	7	6	4	4	3
WI	23	25,953	698	1844	6	9	7	1	0
WV	77	177,573	574	5091	15	22	19	16	5
WY	17	22,401	329	5094	2	7	4	2	2

States are identified by standard two-letter abbreviations.

### Semantic categories

The results of the semantic grouping process, including the semantic categories and their assigned words, are shown in Supplementary Table 1 in the Supplementary Materials.

**Fig. 1:**
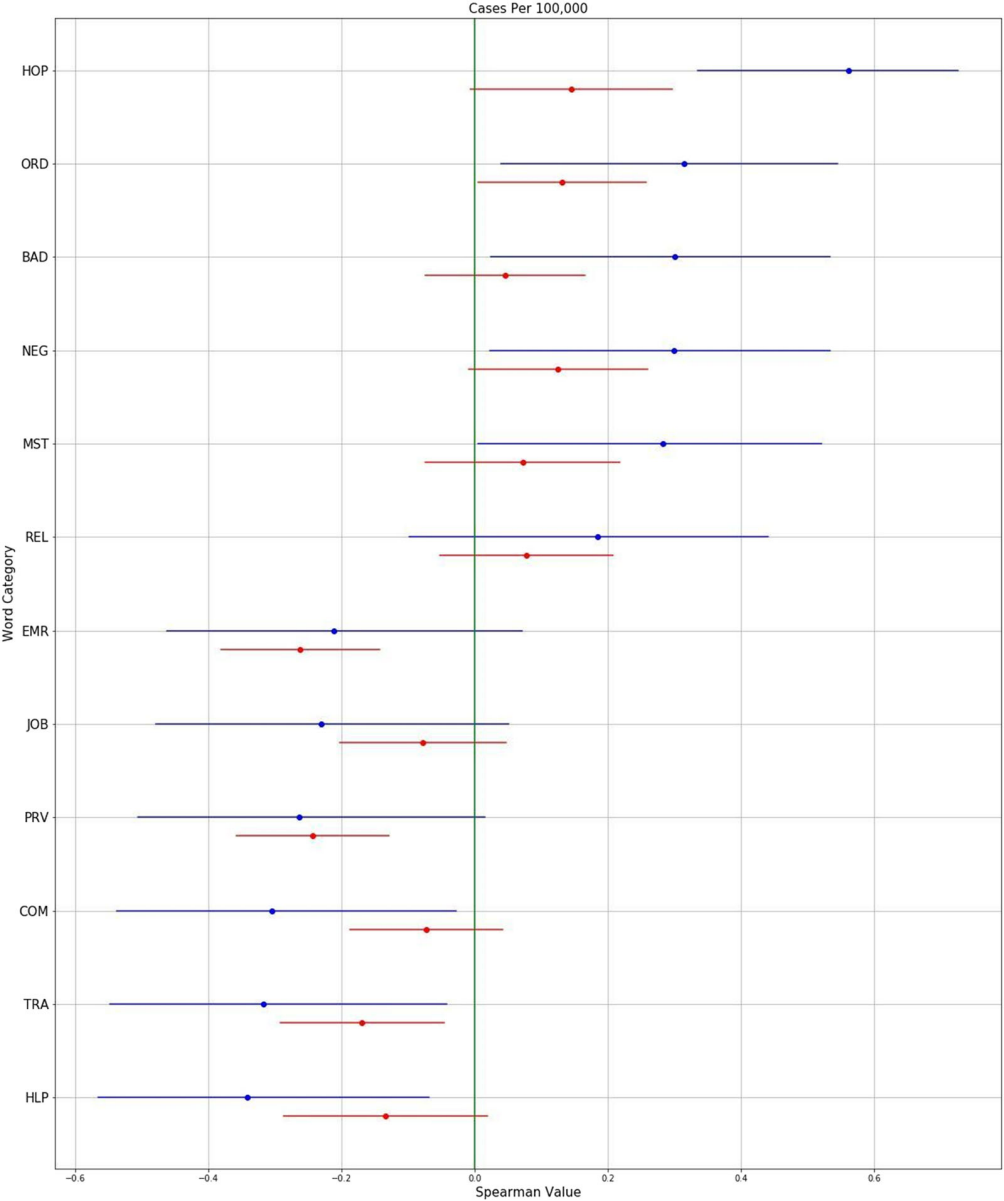
Semantic categories associated with COVID-19 case rates. Semantic categories associated with Covid-19 case rates over both space and time. Average Spearman Rho correlations with 95% confidence intervals, calculated over both space (blue) and time (red). (HOP: Hospital-related; ORD: Strict instructions; BAD: Descriptive bad; NEG: Negation; MST: Extreme descriptive; REL: Religious; EMR: Emergency; JOB: Job-related; PRV: Preventative measures; COM: Formal communication; TRA: Travel-related; HLP: Help and assistance.).

**Fig. 2:**
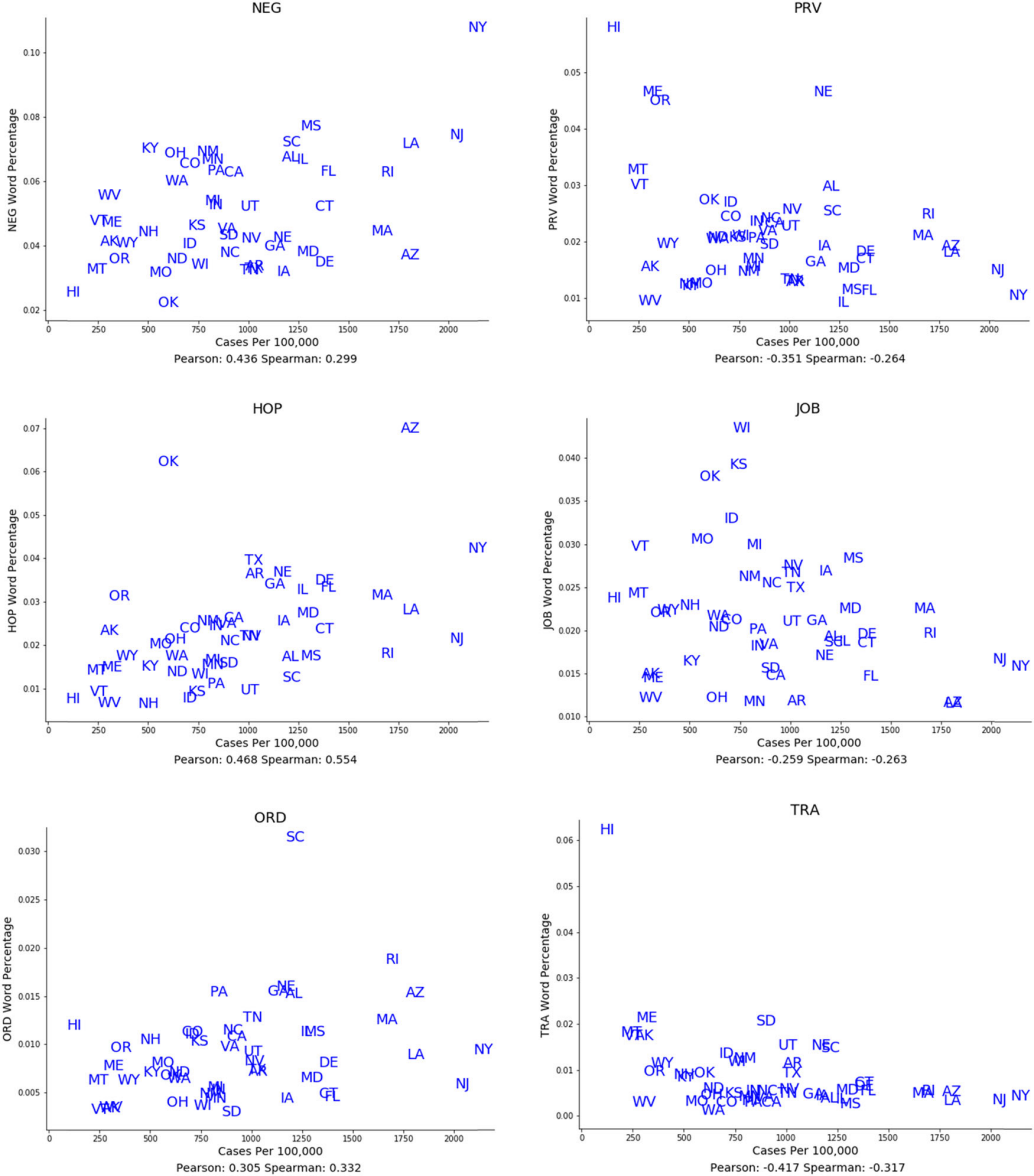
Spatial associations of semantic categories with COVID-19 case rates. Both positive and negative associations are shown. States are represented by standard two-letter abbreviations. (NEG: Negation; JOB: Job-related; HOP: Hospital-related; PRV: Preventative measures; ORD: Strict instructions; TRA: Travel-related.).

**Fig. 3:**
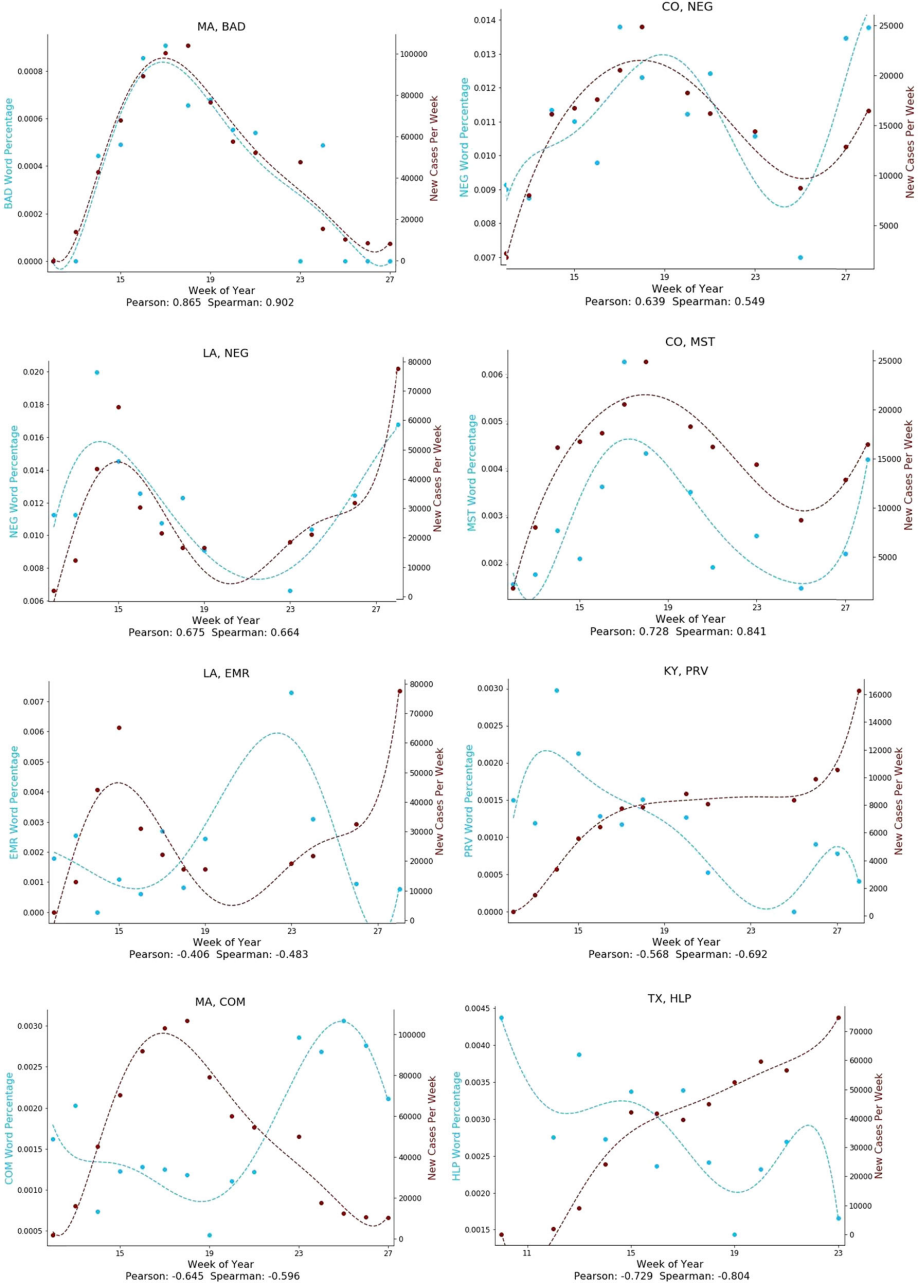
Temporal associations of semantic categories with COVID-19 case rates. Both positive and negative associations are shown. States are represented by standard two-letter abbreviations. (BAD: Descriptive bad; NEG: Negation; MST: Extreme descriptive; EMR: Emergency; PRV: Preventative measures; COM: Formal communication; HLP: Help and assistance.).

Figure 1 shows the average Spearman correlations for the semantic categories that had strong associations over both space and time with COVID-19 case rates. Figure 2 illustrates some of these relationships over space as scatterplots, while Fig. 3 illustrates some of these relationships over time as temporal graphs.

COVID-19 cases rates were positively associated with words relating to hospitals such as “ICU” and “ventilators” (Semantic category “HOP”; Spatial Spearman’s Rho 0.56 [95% CI: 0.33 to 0.75]; Temporal Spearman’s Rho 0.15 [−0.01 to 0.3]). They were also positively associated with negation words such as “can’t” and “no” (NEG; 0.28 [0.02 to 0.52]; 0.07 [−0.01 to 0.26]), words relating to issuing strict public guidance such as “prohibited” and “compliance” (ORD; 0.31 [0.04 to 0.54]; 0.13 [0.00 to 0.26]), descriptive words relating to the concept *bad* such as “terrible” and “worst” (BAD; 0.3 [0.02 to 0.53]; 0.046 [−0.07 to 0.17]), words relating to religion such as “pray” and “God” (REL; 0.19 [-0.1 to 0.44]; 0.08[-0.05 to 0.21]), and words relating to extreme descriptions such as “dramatically” and “extraordinarily” (MST; 0.28 [0.01 to 0.52]; 0.07 [−0.07 to 0.22]).

COVID-19 cases rates were negatively associated with words relating to jobs such as “employment” and “workers” (JOB; −0.23 [−0.47 to 0.06]; −0.08 [−0.2 to 0.05]), words related to travel such as “tourism” and “hotels” (TRA; −0.32 [−0.55 to −0.04]; −0.17 [−0.29 to −0.05]), words describing formal communication formats such as “announcement” and “declaration” (COM; −0.3 [−0.54 to −0.03]; −0.7 [−0.19–0.04]), and words describing helpful actions such as “hospitality” and “assistance” (HLP; −0.34 [−0.57, to −0.07]; −0.13 [−0.29 to 0.02]). Perhaps unexpectedly, words describing emergency situations such as “crisis” and “disaster” (EMR; −0.21 [−0.46 to 0.07]; −0.26 [−0.38 to −0.14]), as well as words describing specific protective measures such as “sanitizer” and “quarantine” (PRV; −0.26 [−0.51 to 0.02]; −0.24 [−0.36 to −0.13]) were negatively correlated with COVID-19 case rates.

### Parts of speech

**Fig. 4:**
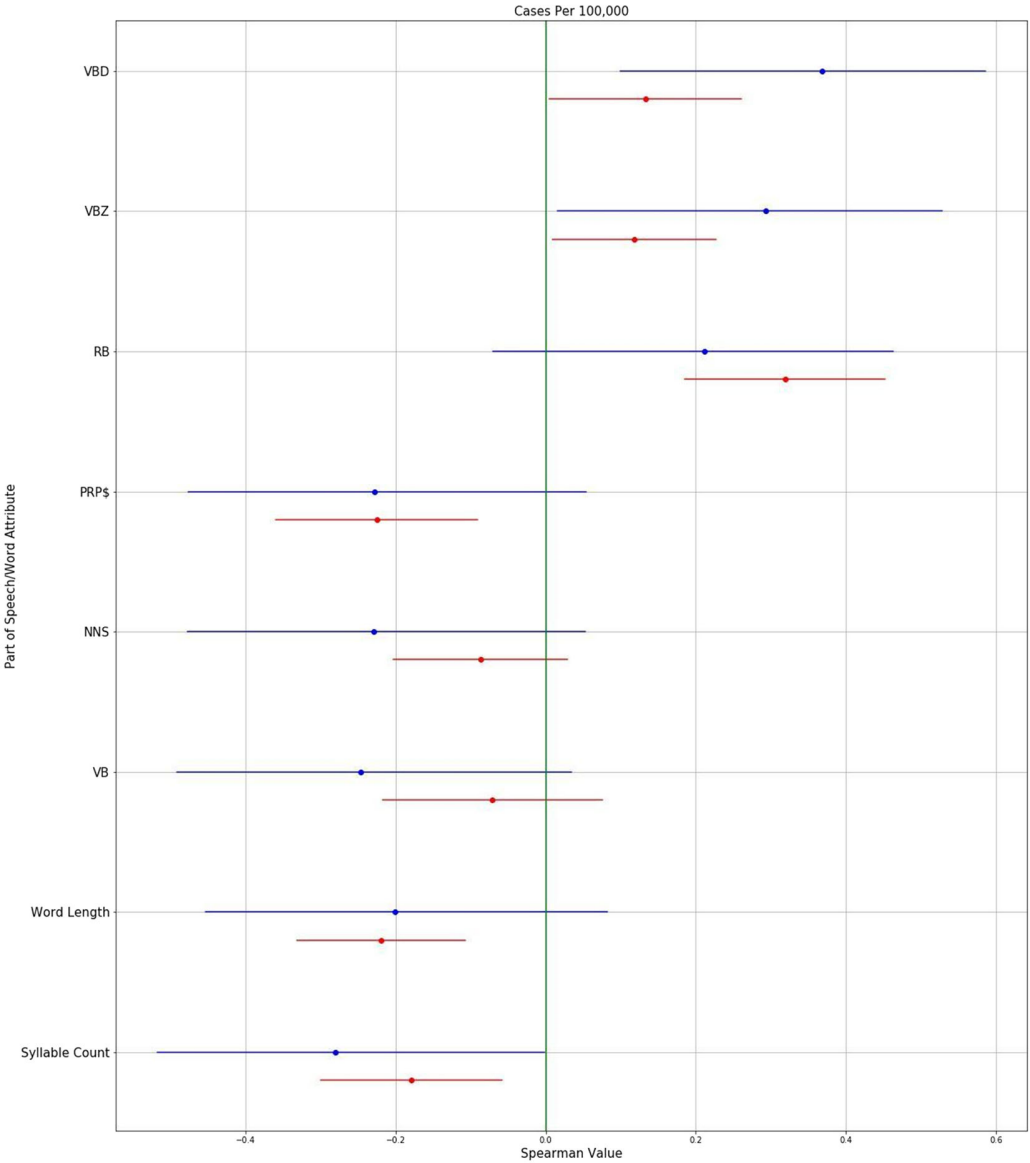
Parts of Speech and linguistic complexity measures associated with COVID-19 case rates. Parts of speech and linguistic complexity measures associated with Covid-19 case rates over both space and time. Average Spearman Rho correlations with 95% confidence intervals, calculated over both space (blue) and time (red). (VBD: Verb, past tense; VBZ: Verb, present tense; RB: Adverb; PRP$: Pronoun, possessive; NNS: Noun, common, plural; VB: Verb, base form).

**Fig. 5:**
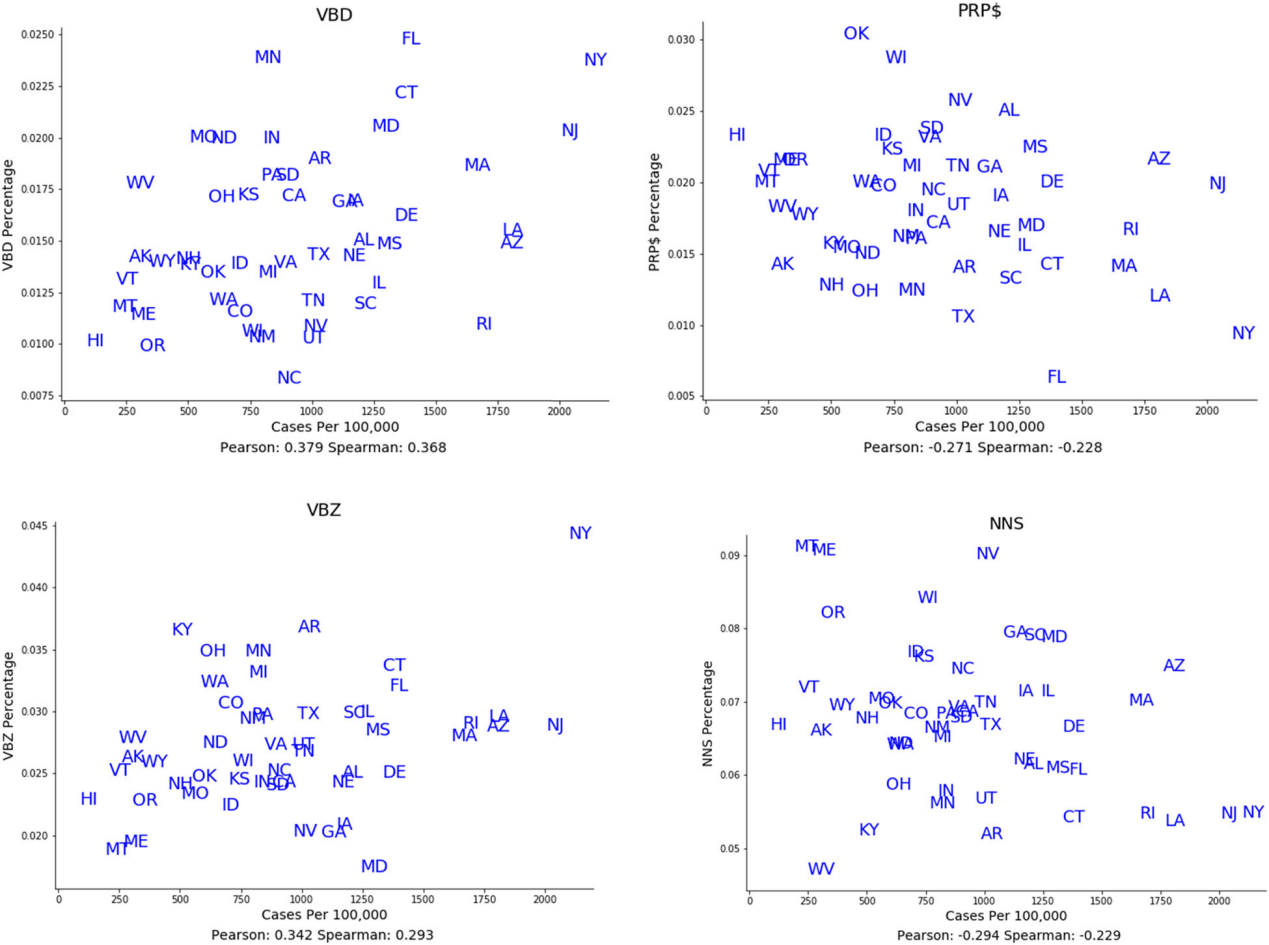
Spatial associations of parts of speech with COVID-19 case rates. Both positive and negative associations are shown. States are represented by standard two-letter abbreviations. (VBD: Verb, past tense; VBZ: Verb, present tense; PRP$: Pronoun, possessive; NNS: Noun, common, plural.).

**Fig. 6:**
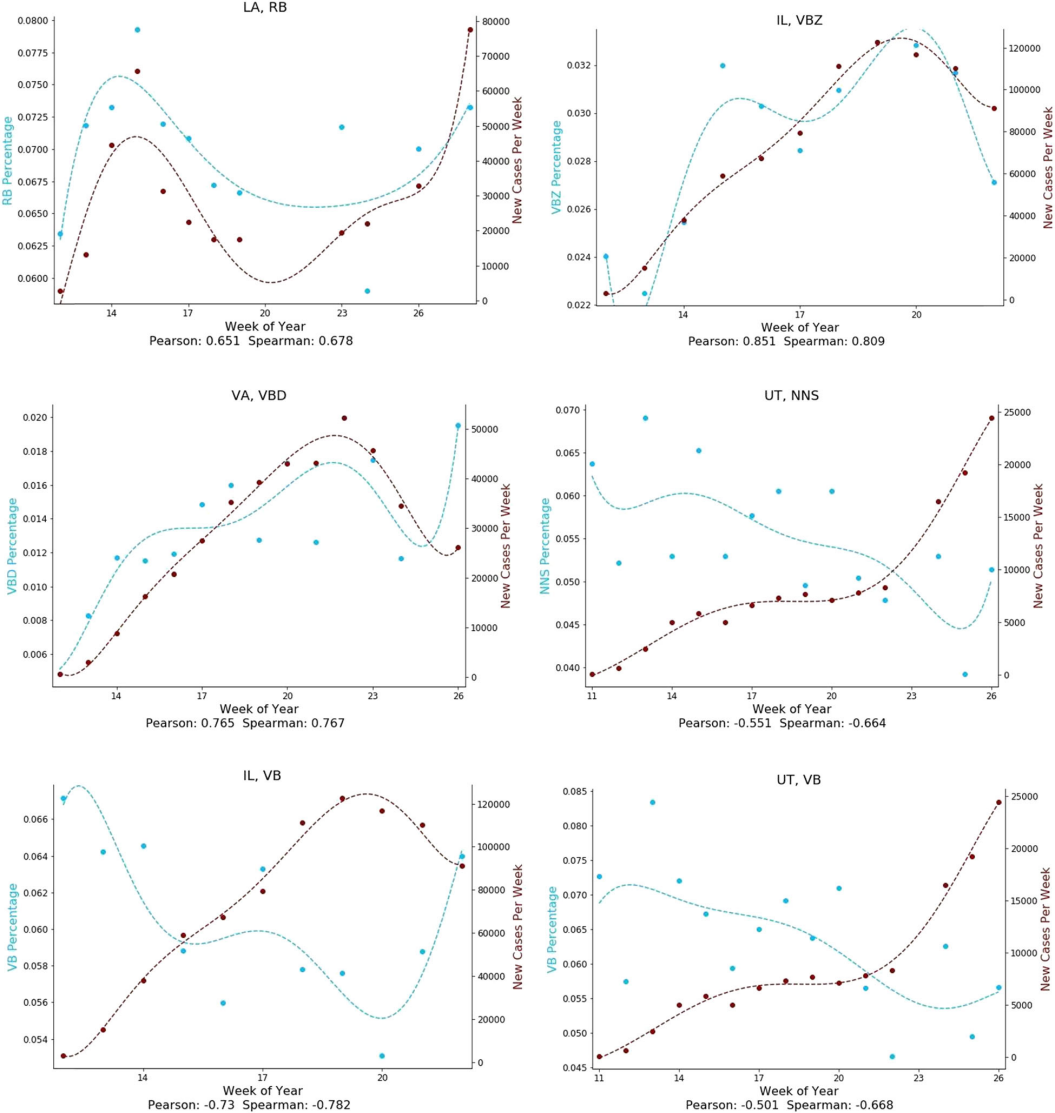
Temporal associations of parts of speech with COVID-19 case rates. Both positive and negative associations are shown. States are represented by standard two-letter abbreviations. (VBD: Verb, past tense; VBZ: Verb, present tense; RB: Adverb; NNS: Noun, common, plural.).

Next, we systematically analyzed all parts of speech for association with COVID-19 case rates over space and time. Figure 4 shows the average Spearman correlations for parts of speech that had strong associations over both space and time with COVID-19 case rates. Figure 5 illustrates some of these relationships over space as scatterplots, and Fig. 6 illustrates some of these relationships over time as temporal graphs.

COVID-19 case rates were positively associated with past-tense verbs such as “asked” and “did” (VBD; 0.13 [0.00 to 0.26]; 0.37 [0.1 to 0.57]), present-tense verbs such as “argues” and “claims” (VBZ; 0.12 [0.01 to 0.23]; 0.29 [0.02 to 0.53]), and adverbs such as “rapidly” or “reliably” (RB; 0.32 [0.19 to 0.45]; 0.21 [−0.07–0.46]).

COVID-19 case rates were negatively associated with possessive pronouns such as “his” or “your” (PRP$; −0.23 [−0.36 to −0.09]; −0.23 [−0.48 to 0.05]), plural nouns such as “people” or “cases” (NNS; -0.09 [−0.2 to 0.03]; −0.23 [−0.48 to 0.05]), and base-form verbs such as “respond” or “walk,” which are typically used in future tense settings (VB; −0.07 [−0.22 to 0.08]; −0.247 [−0.49 to 0.03]).

### Word length/syllable count

**Fig. 7:**
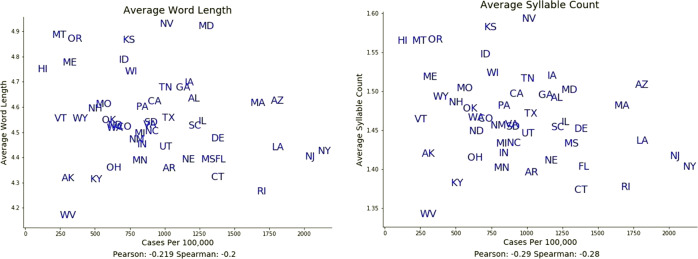
Spatial associations of linguistic complexity measures with COVID-19 case rates. Both word length and syllable count were negatively associated with COVID-19 case rates over space. The drop in word length and syllable count is particularly noticeable in states with the highest COVID-19 case rates. States are represented by standard two-letter abbreviations.

**Fig. 8:**
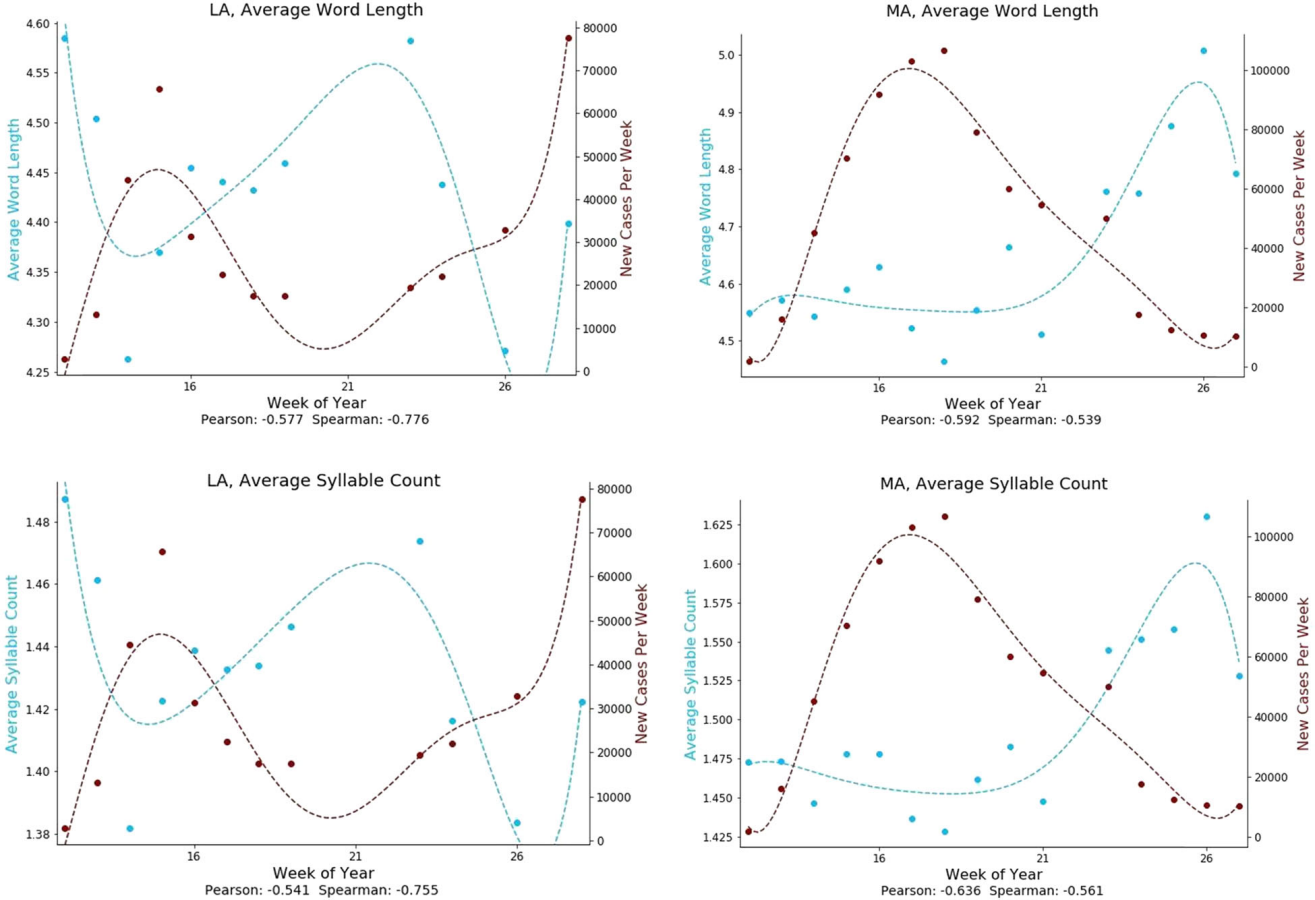
Temporal associations of linguistic complexity measures with COVID-19 case rates. Both word length and syllable count were negatively associated with COVID-19 case rates over time. States are represented by standard two-letter abbreviations.

COVID-19 case rates were negatively associated with average word length and with average syllable count, over both space and time, as shown in Figs. 4, 7, and 8. This negative association between linguistic complexity and COVID-19 case rates was strongest in states experiencing the highest COVID-19 case rates.

## Discussion

By analyzing over 1500 pandemic-related speeches delivered by all 50 US governors, we found that COVID-19 case rates were strongly associated, both spatially and temporally, with the linguistic properties of governor’s speeches, including semantic categories, parts of speech, word length and syllable count. Below we present possible interpretations as to how these results might be understood, recognizing that other possible interpretations also exist. For example, governors may be less influenced by changing cases and more influenced by the evolving political discourse around the pandemic, which could affect their choice of words.

Some of the positive relationships identified were not surprising; for example, the increased use of hospital-related words (HOP), extreme descriptions (MST) and words related to the concept “bad” (BAD) as COVID-19 case rates increased. For example, regarding HOP, New York governor Andrew Cuomo said, “There was a global rush for ventilators and literally we have people on the ground in China.” California Governor Gavin Newsome said, “that represents a 10.7% increase over yesterday.. hospitalizations in the state of California and ICU beds again those are the numbers that I look at first thing every morning.” As cases increased, governors also used stricter words to issue public guidance (ORD), consistent with increasing urgency to promote compliance with public health guidelines.

Some of the negative relationships were also not surprising: As COVID-19 case rates went up, governors spoke less about travel (TRA); travel activity slowed dramatically due to closures and lockdowns, and the relative importance of travel may have decreased in the face of an ongoing public health emergency. As COVID-19 case rates rose, governors also spoke less about words relating to jobs and employment (JOB); while increased pandemic-related unemployment was certainly an important issue, its relative importance may have temporarily decreased in the face of the ongoing pandemic.

We observed a positive relationship between COVID-19 case rates and words focused on religion (REL). During the initial peak of the pandemic, when many people were falling ill and dying, governors often turned to religious and prayer-related terminology to console, show empathy, or offer hope to their constituents. For example, Governor Phil Murphy of New Jersey said, “We keep each of their memories and their families in our prayers, and please join us in that regard.” Governor Jim Justice of West Virginia said, “Only God above knows, and he’ll get us through this. I absolutely am confident about that beyond belief.”

We also observed a negative association between COVID-19 case rates and words describing formal communication formats, such as “announcement” and “declaration” (COM). This is consistent with governors spending less time discussing the formalistic structure and format of their briefings, and more time focused on delivering actionable messages. We also observed a negative association between words describing helpful acts (HLP) and COVID-19 case rates, which may be attributable to governors focusing on telling people to stay in their homes rather than go out and help others.

We also observed some potentially unexpected relationships. There was a negative association between COVID-19 case rates and words describing the situation in terms relating to “emergency” (EMR), consistent with governors not wanting to facilitate further panic as the crisis worsened. We also observed a negative relationship between words relating to preventative measures (PRV) and COVID-19 case rates, whereas one might have expected that governors would increasingly emphasize preventative measures as cases rose. For example, early on, Massachusetts governor Charlie Baker said, “please help us stay ahead of the virus and prevent the spread through the simple steps we have talked about before: face coverings, hand-washing, hygiene, and social distance.” One possible interpretation is that governors continued to speak about preventative measures, but also spoke more about other topics, causing the percentage of words related to preventative measures to decrease.

While there was much discussion early in the pandemic around testing, personal protective equipment, and the status of the elderly, particularly in nursing homes, we did not find any strong correlations for these categories.

As COVID-19 case rates increased, we found an overall shift in verb usage from future tense (VB) to past (VBD) and present tenses (VBZ). This is consistent with governors shifting from talking about what they are planning to do in anticipation of the looming crisis, to describing what they are currently doing or what they have already done to respond to increasing case rates.

We observed a shift from nouns to verbs and adverbs as COVID-19 case rates rose. This is consistent with speeches becoming more oriented towards describing actions being taken to respond to the pandemic. Additionally, there was a shift away from the use of personal pronouns (PRP$), consistent with governors speaking more about what is being done rather than by whom it is being done.

We observed a strong, positive relationship between COVID-19 case rates and the use of negation words (NEG), consistent with the interpretation that as case rates rose and pressure to respond to the crisis mounted, governors responded in a number of ways that involved the use of negation words. These included speaking in a defensive manner about the actions they were taking and the limits of their authority, as well as highlighting the prevailing uncertainty that they faced. For example, Governor John Bel Edwards of Louisiana said, “I don’t know when that’s going to come and I don’t know in what amounts, I don’t want to speculate beyond saying that at some point in the next couple of weeks we should have an REC meeting.” New York governor Andrew Cuomo said, “I can’t mandate personal behavior, I never could. My strategy from day one, knowing that we were going to have to ask people to do things that no government has asked them to do, maybe since World War I or World War II.” Mississippi governor Tate Reeves said, “I don’t have the authority to shut them down, therefore I don’t have the authority to reopen them. Mississippi is not China but we have to continue to be vigilant in attacking this virus.” West Virginia governor Jim Justice said, “We don’t need to hear that that’s nothing but garbage. We don’t need to hear that we know what this killer is all about and it’s everywhere, and on the other side we really don’t need to hear all the noise that says, you know, really and truly, now we’re opening up swimming pools so we’re going to kill 19 other people and everything we got to know we don’t need that noise either.”

We also observed a negative relationship between COVID-19 case rates and average word length and syllable count. Interestingly, this association was strongest in US states that experienced the highest rates of COVID-19 cases. The use of shorter, simpler words as COVID-19 case rates surged is consistent with a characteristic response to stress on the part of the governors. Public speaking tasks have been shown to be highly associated with acute physiological stress^20^. Saslow et al. found a decrease in linguistic complexity associated with stress, along with elevated heart rate and increased cortisol reactivity^21^. Buchanan et al. found decreases in word productivity to be associated with stress, increased cortisol levels and elevated heart rate^22^. Acute stress responses to stress-inducing speech tasks have been found to reduce function of the prefrontal cortex^23^, decrease cognitive flexibility^24^, and impair working memory^25^ (see excellent review by Saslow et al.^21^).

Some studies have looked more broadly at how leaders respond to crises. Suedfeld and colleagues found that integrative complexity of speeches by political^17^ and academic leaders^18^ declined during times of crisis^19^. Green et al. studied communication by US Congress members during the COVID-19 pandemic and identified increasing polarization between political parties during the start of the pandemic^26^. Pennebaker and Lay studied 35 of Rudy Giuliani’s press conferences over years that included two periods of crisis, including the September 11, 2001 attacks; they found certain linguistic shifts associated with crisis, including the use of negation words and shorter words, though not all shifts were consistently observed over both periods of crisis^9^.

To the best of the authors’ knowledge, the present study is the first to identify the spatial and temporal relationships between pandemic dynamics and pandemic-related public communication by leaders, including examining characteristic linguistic measures of stress in response to crisis intensity. These associations can be used to guide and inform future research about the impact of the linguistic properties of political leaders’ speech on important public health communication measures such as compliance by members of the public.

This study is subject to certain limitations. Though we collected over 1500 speeches containing over 4 million words from all 50 US governors, some delivered speeches were not available to be included in the analysis. Whereas we analyzed the response to a major pandemic within one level of government of one large country, responses to other crises in other locations and other levels of government may vary. We analyzed speeches at the single-word level, providing many rich dimensions of information; multi-word patterns contain further information, and are a subject for future studies. Whereas we focused on speeches during the first few months of the pandemic, responses during later stages of the pandemic may differ. The spatial analyses comparing all 50 states may be subject to potential confounding, as certain non-pandemic-related state variables such as population density and political affiliation may affect word choice. To address this, we also conducted the temporal analyses—case rates and certain linguistic features were found to move together over time as cases rose and then fell within multiple locations, eliminating many potential confounding variables (e.g., population density) which do not change significantly on the timescale of a few weeks.

As mentioned above, the content and linguistic properties of governor’s speeches may be influenced by political approach and priorities, personal demeanor and the choice of which information sources a governor chooses to rely on. Governors may also deliberately modify their speech styles to increase comprehension by general audiences. In this study, we have proposed possible interpretations describing how COVID-19 case counts may affect governor speech patterns. It may also be the case that governors’ speeches may affect COVID-19 case counts. This is another topic worthy of future study.

Computational linguistic methods are a powerful tool for exploring how leaders respond to emerging crises. By assembling and analyzing a large corpus of speeches delivered by all 50 US state governors during the initial months of the COVID-19 pandemic, we found that governor speech patterns were strongly associated with COVID-19 case rate dynamics across both space and time. Several of the observed effects were consistent with responses to increased stress at the height of one of the largest public health crises in modern times. Such effects may serve to decrease the quality and impact of public health communication, or alternatively, may serve to improve it (as shorter words and greater urgency may heighten the power of the message). Analysis of similar bodies of text from other political leaders and public figures, at different levels of government including at the national level, from different public crises, and from different stages of the COVID-19 pandemic would be key to understanding if the patterns identified here are consistent across different conditions. It would also be worthwhile to study additional potential markers of stress, and to apply more advanced NLP methods. Investigating and understanding the effects of these characteristic stress responses is important for improving communication during major public health crises, both present and future.

## Methods

### Data collection

We collected pandemic-related speeches delivered by governors of all 50 US states from February 27, 2020 through July 14, 2020, a period during which most states experienced at least one wave of COVID-19 cases. Transcripts of public speeches were obtained from four primary sources: (1) Governors’ offices, via public websites or direct correspondence; (2) the commercial transcription service *Rev*; (3) the online video sharing service *YouTube*, and (4) the social networking service *Facebook*. All transcripts were curated to include only words spoken by the governors themselves. There was no apparent bias in which speeches were made available, and speeches from the governors’ offices do not appear to have been redacted in any way.

For each of the 50 US states, we obtained data on confirmed cases of COVID-19 per 100,000 persons, assembled by the *New York Times* based on data published by state and local health authorities across the United States^27^.

### Data analysis

For each state, for each word, we tabulated total word counts for each speech. We included only words spoken by 20 or more governors, and which represented at least 0.02% of all words spoken across all speeches in at least one of the 50 US states.

Words that met the above criteria were grouped by two independent raters into semantic categories. Words with multiple common meanings, or that could not be neatly categorized into a single semantic category, were excluded from the semantic grouping analysis on a case-by-case basis.

In addition to grouping words by semantic category, we also grouped words by part of speech using the part of speech tagger from the *Natural Language Toolkit* library of Python version 3.6.2. We also calculated measures of linguistic complexity, including average word length (number of letters) per speech, and average word syllable count per speech.

We systematically analyzed the associations of each of these linguistic features (semantic categories, parts of speech, word length and syllable count) with COVID-19 case rates per 100,000 persons. We analyzed these associations *over space*—across each of the 50 US states for the entire duration of the study period, as well as *over time*—across each week of the study period for a given single US state. For the spatial analyses, we normalized word counts as a percentage of all words spoken by that governor across all speeches in that state. For the temporal analyses, we normalized word counts as a percentage of all words spoken by that governor across all included speeches in that state during that given week. To ensure sufficient sample size, the temporal analyses were conducted only in states for which a total of at least 50,000 words from speeches were available.

For the spatial analyses, we calculated confidence intervals using the formula for finding the confidence interval around a single Spearman’s Rho value: where *r* is the estimate of the correlation and *n* is the sample size. For the temporal analyses, in which multiple Spearman’s Rho values were available (one from each included state), we used the standard formula for a distribution of values. As a visual aid, we fit polynomial curves to the temporal plots using the *numpy.polyfit* function of Python version 3.6.2, and plotted them alongside the original data points.

### Reporting summary

Further information on research design is available in the Nature Research Reporting Summary linked to this article.

## Supplementary information

Supplementary Materials

Reporting Summary

## Data Availability

The data used in this study are available upon request from the authors.

## Code availability

The code used in this study is available upon request from the authors.
