# Supplementary material for: The language of crisis: spatiotemporal effects of COVID-19 pandemic dynamics on health crisis communications by political leaders
Source: NPJ Digit Med. 2022 Jan 10;5:1. doi: 10.1038/s41746-021-00554-w (PMC8748550; doi:10.1038/s41746-021-00554-w)
Supplement: Supplementary file 1 — Supplementary Materials [file 41746_2021_554_MOESM1_ESM.pdf]

Supplementary Materials

The Language of Crisis: Spatiotemporal Effects of COVID-19 pandemic dynamics on health crisis communications by political leaders

Benjamin J. Mandl [1,2] , Ben Y. Reis, PhD [1,3]

[1] *Predictive Medicine Group, Computational Health Informatics Program, Boston Children's Hospital, Boston, MA*

[2] *Washington University, St. Louis, MO*

[3] *Harvard Medical School, Boston, MA*

Supplementary Table 1. Semantic categories, along with their assigned words.

Category	Word
BAD	bad
BAD	serious
BAD	terrible
BAD	worse
BAD	worst
CLO	close
CLO	closed
CLO	closing
CLO	closure
CLO	closures
CLO	shut
COLLAB	collaboration
COLLAB	collaborative
COLLAB	cooperation
COLLAB	partners
COLLAB	partnership
COLLAB	partnerships
COM	announce
COM	announced
COM	announcement

COM	announcements
COM	announcing
COM	briefing
COM	briefings
COM	communication
COM	declaration
COM	declared
COM	explain
COM	message
COM	outreach
COM	update
COM	updated
COM	updates
CS	corona
CS	coronavirus
CS	cova
CS	covet
CS	covid
CS	covid-19
CS	covid19
CS	epidemic
CS	kovat
CS	pandemic

CS	virus
DEAD	deadly
DEAD	death
DEAD	deaths
DEAD	died
DEAD	fatalities
DISC	conversation
DISC	conversations
DISC	discuss
DISC	discussion
DISC	discussions
DISC	hear
DISC	heard
DISC	listen
DISC	listening
DISC	speak
DISC	speaking
DISC	spoke
DISC	talked
DISC	talking
DISC	tell
DISC	told
DOWN	decline

DOWN	declining
DOWN	down
DOWN	downward
DOWN	falls
DOWN	reduce
DOWN	reducing
EC	commerce
EC	consumer
EC	consumers
EC	customers
EDU	classroom
EDU	college
EDU	education
EDU	school
EDU	schools
EDU	student
EDU	students
EDU	teacher
EDU	teachers
EDU	universities
EDU	university
EG	budget
EG	fiscal

EG	fund
EG	funding
EG	funds
EG	ppp
EG	spend
EG	tax
EG	taxes
EMR	battle
EMR	crisis
EMR	critical
EMR	dangerous
EMR	disaster
EMR	emergency
EMR	fight
EMR	severe
EMR	threat
EMR	vital
ET	business
ET	businesses
ET	commercial
ET	companies
ET	dollar
ET	economic

ET	economy
ET	finance
ET	financial
ET	industries
ET	industry
ET	infrastructure
ET	inventory
ET	investment
ET	loan
ET	loans
ET	manufacturing
ET	market
ET	money
ET	production
ET	purchase
ET	sales
FEAR	afraid
FEAR	anxiety
FEAR	anxious
FEAR	fear
FEAR	worry
G	administration
G	agencies

G	agency
G	congress
G	constitution
G	council
G	department
G	departments
G	executive
G	federal
G	gov
G	government
G	governments
G	jurisdictions
G	legislation
G	legislative
G	legislature
G	municipal
G	regulatory
G	state
G	state's
G	states
G	washington
GA	camps
GA	concerts

GA	conference
GA	congregate
GA	crowds
GA	events
GA	gather
GA	gatherings
GA	sports
GOOD	amazing
GOOD	excellent
GOOD	good
GOOD	great
GOOD	incredible
GOOD	incredibly
GOOD	wonderful
GP	commissioner
GP	congressional
GP	governor
GP	governor's
GP	governors
GP	leaders
GP	legislators
GP	mayor
GP	mayors

GP	officials
GP	president
GP	president's
GP	representatives
GP	senator
GP	trump
GP	vice
HARD	challenge
HARD	challenges
HARD	challenging
HARD	difficult
HARD	overwhelmed
HARD	overwhelming
HARD	problem
HARD	problems
HARD	struggling
HARD	tough
HLP	assist
HLP	assistance
HLP	care
HLP	cares
HLP	compassion
HLP	donate

HLP	donated
HLP	help
HLP	helped
HLP	helpful
HLP	helping
HLP	helps
HLP	hospitality
HLP	relief
HLP	supporting
HLP	volunteer
HO	construction
HO	evictions
HO	homeless
HO	homes
HO	housing
HO	rent
HO	shelter
HOP	bed
HOP	beds
HOP	clinics
HOP	hospital
HOP	hospitalization
HOP	hospitalizations

HOP	hospitalized
HOP	hospitals
HOP	icu
HOP	intensive
HOP	patients
HOP	surgeries
HOP	surgical
HOP	ventilator
HOP	ventilators
HOPE	hope
HOPE	hopeful
HOPE	hopefully
HOPE	hoping
HOPE	optimistic
HPG	gear
HPG	gloves
HPG	gowns
HPG	mask
HPG	masks
HPG	ppe
HPG	shields
HPR	doctor
HPR	doctors

HPR	dr
HPR	nurses
HPR	physicians
IN	bar
IN	bars
IN	gyms
IN	indoor
IN	restaurant
IN	restaurants
IN	salons
JOB	employees
JOB	employer
JOB	employers
JOB	employment
JOB	job
JOB	jobs
JOB	labor
JOB	self-employed
JOB	unemployed
JOB	unemployment
JOB	workers
JOB	workforce
JOB	workplace

MST	biggest
MST	dramatically
MST	entire
MST	extraordinary
MST	highest
MST	historic
MST	huge
MST	largest
MST	leading
MST	tremendous
MST	unprecedented
MST	very
NEG	aren't
NEG	can't
NEG	cannot
NEG	didn't
NEG	doesn't
NEG	don't
NEG	hadn't
NEG	haven't
NEG	isn't
NEG	never
NEG	no

NEG	not
NEG	nothing
NEG	shouldn't
NEG	wasn't
NEG	won't
NEG	wouldn't
NEG	wrong
OPE	easing
OPE	lifting
OPE	open
OPE	opened
OPE	opening
OPE	reopen
OPE	reopened
OPE	reopening
ORD	command
ORD	compliance
ORD	demand
ORD	enforce
ORD	enforcement
ORD	instruction
ORD	mandatory
ORD	prohibited

ORD	regulations
ORD	rules
ORD	stay-at-home
OUT	beach
OUT	beaches
OUT	fishing
OUT	outdoor
OUT	outside
OUT	parks
OUT	recreation
OUT	takeout
OUT	walk
P	campaign
P	elected
P	election
P	elections
P	political
P	politics
P	republican
P	vote
PRG	prepare
PRG	prepared
PRG	preparing

PRG	prevent
PRG	prevention
PRG	vigilant
PRO	he
PRO	he's
PRO	her
PRO	him
PRO	his
PRO	i
PRO	i'd
PRO	i'll
PRO	i'm
PRO	i've
PRO	me
PRO	mine
PRO	ourselves
PRO	she
PRO	she's
PRO	their
PRO	themselves
PRO	they
PRO	they'll
PRO	they're

PRO	they've
PRO	we
PRO	we'd
PRO	we'll
PRO	we're
PRO	we've
PRO	who's
PRO	who've
PRO	you
PRO	you'll
PRO	you're
PRO	you've
PRO	your
PRO	yourself
PRV	distance
PRV	distancing
PRV	hand
PRV	hands
PRV	hygiene
PRV	isolate
PRV	physical
PRV	physically
PRV	quarantine

PRV	sanitizer
PRV	wash
PRV	washing
REL	church
REL	churches
REL	god
REL	pray
REL	prayer
REL	prayers
REL	religious
REL	worship
S	antibody
S	cdc
S	fda
S	lab
S	laboratory
S	labs
S	model
S	modeling
S	models
S	reagents
S	research
S	science

S	scientific
S	study
S	vaccine
SP	case
SP	cases
SP	contagious
SP	contracted
SP	exposed
SP	exposure
SP	hotspots
SP	infected
SP	infection
SP	infections
SP	outbreak
SP	outbreaks
SP	spread
SP	spreading
SP	transmission
SPEED	quick
SPEED	quickly
SPEED	rapidly
SPEED	rush
SUG	encourage

SUG	guidance
SUG	guidelines
SUG	precautions
SUG	recommendations
SUG	recommended
SUG	suggestions
SY	asymptomatic
SY	fever
SY	symptom
SY	symptomatic
SY	symptoms
T	diagnostic
T	screen
T	screening
T	swabs
T	test
T	tested
T	testing
T	tests
THX	appreciate
THX	appreciation
THX	grateful
THX	gratitude

THX	thank
THX	thankful
THX	thanks
TRA	hotels
TRA	tourism
TRA	travel
TRA	traveling
TRA	trips
TRACK	indicator
TRACK	indicators
TRACK	monitor
TRACK	monitoring
TRACK	surveillance
TRACK	tracing
TRACK	track
TRACK	tracking
TRACK	watching
UP	growth
UP	increase
UP	increased
UP	increasing
UP	raise
UP	rise

UP	up
UP	uptick
